# Through the Calf’s Eye: Exploring Infrared Thermography to Uncover Pair-Housed Calves’ Affective States

**DOI:** 10.3390/ani16020182

**Published:** 2026-01-08

**Authors:** Gaia Pesenti Rossi, Sara Barbieri, Emanuela Dalla Costa, Michela Minero, Elisabetta Canali

**Affiliations:** Department of Veterinary Medicine and Animal Sciences, University of Milan, 26900 Lodi, Italy; sara.barbieri@unimi.it (S.B.); emanuela.dallacosta@unimi.it (E.D.C.); michela.minero@unimi.it (M.M.); elisabetta.canali@unimi.it (E.C.)

**Keywords:** pair housing, infrared thermography imaging, affective states, lateralization, ocular thermal asymmetry

## Abstract

Pair housing has been proven to improve calves’ welfare during the pre-weaning phase, potentially promoting positive emotions. Based on the emotional valence hypothesis, according to which positive emotions are mainly processed in the left brain hemisphere, infrared thermography (IRT) may detect changes in eye temperature, particularly thermal asymmetries. This study aimed to evaluate whether pair-housed calves presented ocular thermal asymmetry, compared to individually housed calves during the pre-weaning phase. Fifty-six Friesian female calves from two commercial dairy farms were enrolled and housed either individually or in pairs from birth until 8 weeks of age. IRT images of the lacrimal caruncle of both eyes were collected at 7, 21, 35, and 56 days of age. A linear mixed model tested the effects of housing, farm, year, and timepoint. No significant effect of pair housing on ocular asymmetry emerged, while absolute eye temperatures were significantly higher in pair-housed calves. Moreover, eye temperature declined over time, suggesting reduced arousal and habituation with age. Although the lateralization hypothesis was not confirmed, the study offers insights into IRT for assessing calf emotions and supports further investigation in positive contexts to better explore links between housing, emotional valence, and brain activity.

## Simple Summary

This study investigates whether housing calves in pairs, rather than individually, influences their emotional state, resulting in changes in eye temperature. Eye temperature can be measured with infrared thermography, and it has been suggested that differences between the left and right eye may indicate the processing of emotions of different valences. Fifty-six calves were enrolled in the study and housed either individually or in pairs from birth to eight weeks of age, and eye temperature was recorded at 7, 21, 35, and 56 days of age. Data were analyzed to verify whether housing system affected difference in the temperature of the two eyes and whether overall eye temperature changed over time. The results showed that pair housing did not lead to differences between the left and right eye, suggesting no clear link to brain emotion-processing patterns. However, calves housed in pairs consistently had higher eye temperatures than individually housed calves, and eye temperature gradually decreased as all calves grew older. The study explores the use of thermography imaging for the assessment of calves’ emotions, highlighting the limitations but also the potentialities, and supports further investigation in positive contexts to better explore links between housing, emotional valence, and brain activity.

## 1. Introduction

Emotional states have evolved to guide behavioral responses and decision-making, supporting survival by promoting resource acquisition and damage avoidance [1]. In humans, emotions are defined as category of conscious experiences (“subjective feelings”) that can be reported linguistically and categorized (i.e., “happiness” or “sadness”) and which have a valence (positive or negative) [2]. The human experience of feelings has frequently generated interest on whether non-human animals are able to experience something similar and, if so, how it can affect the quality of their lives [2]. The main difficulty, of course, is that non-human animals cannot verbally report their emotional state. The study of emotions, nevertheless, can comprehend their different components: subjective, neurophysiological, behavioral, and cognitive. For this reason, several approaches have been proposed in the study of animal emotions: among them, one is analyzing “emotion-generating context”. This approach is based on establishing an animal’s emotional state (or “ground truth emotional state”) reasonably linked to an event based on our own human experience and use this to identify indicators of animal emotions [3]. Any threats to an animal’s survival and/or fitness, in fact, can be assumed to produce negative affective states, while any context that improves fitness can be assumed to produce positive states [2]. For example, de Oca et al. [4] considered hot-iron disbudding as a model of negative affective states (linked to pain and restraint) and aimed at investigating facial thermal asymmetries in dairy calves. Facial thermal asymmetries were measured through infrared thermography and analyzed based on the emotional valence hypothesis, where negative emotions are processed more intensively in the right hemisphere and the left hemisphere has more involvement in the processing of positive ones [5,6].

Infrared thermography (IRT) enables the non-invasive, real-time measurement of surface temperatures in animals: thermal cameras, in fact, detect infrared energy emitted from the animal’s external surface, convert it to temperature and display an image of temperature distribution (i.e., the “thermogram”) that is represented as pixels varying in colors or shades for different temperatures [7]. Images are typically taken in highly vascularized areas, known as “thermal window”, such as the eye (i.e., lacrimal caruncle) or ears. This technique allows the detection of physiological and pathological changes in body heat emission in real time and therefore can be applied to the study of thermal patterns associated with the processing of emotions. For instance, alterations in the sympathetic nervous system cause the redirection of blood flow to skeletal muscles, in particular showing temperature fluctuation in the orbital region where there are the rich capillary beds of lacrimal caruncle and posterior border of the eyelids, which are highly innervated by the sympathetic nervous system [8,9,10,11]. This approach has been conducted for studying emotions in cows and calves [10,12,13,14,15]; however, these studies have focused mostly on negative situations, pain, stress, and fear. For example, Stewart and colleagues found that cows showed a drop and a subsequent gradual rise in the temperature of the inner corner of the eye in response to negative handling [13], hot-iron disbudding, and surgical castration [12,15].

In the context of growing interest towards positive welfare, trials should be made also to assess the positive valence of cattle emotions. During the pre-weaning phase, pair housing has been outlined giving significant improvement in the welfare of dairy calves, compared to individual housing [16,17]. In fact, not only has pair housing been associated with improved feed intake and growth performance in pre-weaned calves, particularly when implemented early in life [18,19,20], but it has also been shown to positively influence calves’ behavior and emotional states. Pair-housed calves were found less reactive and fearful, more exploratory in novel environments, and less neophobic toward unfamiliar feeds and conspecifics, indicating improved coping abilities [16,21,22,23,24]. In particular, Bučková et al. [25] reported that pair-housed calves responded more positively to ambiguous cues compared to individually housed calves, suggesting more positive affective states and optimism.

The aim of this study was to explore whether pair housing influences thermal indicators of emotional valence, including lateralization and absolute eye temperatures, detected with infrared thermography and compared to individual housing during the pre-weaning phase.

## 2. Materials and Methods

The protocol of the present study was approved by the Animal Welfare Committee of the University of Milan (OPBA_40_2023).

### 2.1. Animals and Exclusion Criterion

This study took place in two commercial dairy farms in Northern Italy. A total of 56 Friesian female calves were enrolled in the study, with 32 calves from Farm 1 (Calf Barn Indoor), and 24 calves from Farm 2 (Calf Barn Outdoor). Data collection was carried out in two replicates: the first from July to November 2023, and the second from July to September 2024. During each replicate, 8 calves were pair housed and 8 individually housed in farm 1; while in Farm 2, 6 were pair housed and 6 individually housed.

After birth calves were subjected to the farm-standard neonatal care: calves were separated from the mother, and their navel was disinfected with iodine solution. Colostrum was evaluated with Brix refractometer and administrated whether adequate (>22% Brix). Each calf received 4 L colostrum administration within 6 h from birth, through tube-feeding. On the second day of age (timepoint T1), calves’ immunity was evaluated by measuring the Brix% of serum with a hand refractometer, and calves with poor transfer of passive immunity, defined as having Serum %Brix below 8.1% [26], were excluded from the study.

### 2.2. Housing and Management

Calves were managed in compliance with Council Directive 2008/119/EC.

In both farms, calves were housed individually or in pairs from birth until 8 weeks of age (i.e., 55 ± 1 days of age) and allocated to the housing treatment according to the farm birth trend: calves born within 24 h of each other were pair housed, while the others were housed individually. At 55 ± 1 days of age, calves were moved to group pens (15–20 calves per group; 9.50 m × 5.50 m) with an automatic milk feeding system, where they were then weaned at 72–74 days of age in Farm 1 and at 90 days of age in Farm 2.

As previously mentioned, Farm 1 had an indoor system with pens for individual and pair housing, both providing 2.59 m^2^ per calf. Farm 2 was equipped with an outdoor system with individual igloos (2.68 m^2^ per calf) and pair igloos (3.00 m^2^ per calf), both with access to an additional outdoor area (respectively, 1.78 m^2^/individually housed calf and 2.80 m^2^/pair housed calf). Straw was used as bedding in all cases, and additional straw was supplied when necessary.

To assess the feasibility of pair housing in commercial dairy farming system, the usual farm management was maintained throughout the study: in particular, regarding colostrum and milk management, vaccinations, disbudding, and treatment.

Milk replacer was provided twice daily using teat buckets (at approximately 08:00 and 17:00). The quantity progressively increased from 2.5 to 4 L per meal during the first three weeks of life and subsequently remained at this level until eight weeks of age. The composition of the milk replacer diets was slightly different between farms: in Farm 1, the milk replacer contained 22.5% crude protein, 18.0% crude fat, 8.0% crude ash, and no detectable crude fiber; in Farm 2, it contained 27.0% crude protein, 18.0% crude fat, 6.5% crude ash, and 0.2% crude fiber. Calf starter and hay were provided starting from 2 and 7 days of age, respectively.

### 2.3. Health Assessment

The general growth and wellbeing of the calves were monitored daily by trained farm staff. Records included milk intake and the provision of starter feed and hay, along with the assessment of different indicators:

- Vitality score [27]: calves were scored from 5 (alert, stands unassisted) to 1 (permanent lateral recumbency/coma). Intermediate scores reflected quieter behavior with need for stimulation to stand (4), unstable stance after being lifted from sternal recumbency (3), or permanent sternal recumbency with apathy (2);
- Fecal score [28]: 0 = normal; 1 = semi formed/pasty; 2 = loose but remaining on bedding; 3 = watery, penetrating bedding. Diarrhea was defined as a score ≥ 2;
- Ocular discharge [29]: 0 = absent; 1 = presence of wet or dry discharge ≥ 3 cm;
- Nasal discharge [29]: 0 = absent; 1 = dense, visible discharge (transparent to yellow-green);
- Cough: spontaneous occurrence.

Any health issues, disease outbreaks, or treatments were carefully recorded. When clinical signs were evident, the farm veterinarian was promptly notified, and calves were examined and treated accordingly.

At 2, 7, 21, 35, and 56 days of age (named, respectively, T1, T2, T3, T4, and T5), calves underwent a complete clinical examination made by a veterinarian, assessing general appearance and demeanor of animals, including the vitality score (scored according to Boccardo et al. [27]), temperature (measured with a rectal thermometer), fecal consistency (scored according to [28]), mucosal appearance, presence of eventual discharges (ocular/nasal, assessed also with Welfare Quality^®^ scoring system [29]), hydration status according to eye position and eyelid skin tent, auscultation of thorax and abdomen, measurement of heart and respiratory rates, and evaluation of navel and joints. Based on these findings animals were categorized as “healthy” or “sick”.

### 2.4. Thermography Images

At 7, 21, 35, and 56 days of age (T2, T3, T4, and T5, respectively) the temperature (°C) of the eyes (i.e., lacrimal caruncle) of each calf was measured using a handle-infrared camera FLIR E76 (FLIR Systems AB, Täby, Sweden). This area was initially selected based on literature findings [4,12,30], as these areas have been considered both in the assessment of emotions and as indicators of illness and stress. Thermographic images were acquired from both the right and left eyes of each calf, with the order of eye acquisition randomized. Image collection was performed to obtain at least three images of adequate quality per eye for subsequent analyses, resulting in a total of 336 images per timepoint. The thermographic infrared images were captured by a certified technician. The infrared camera has a thermal resolution of 320 × 240 pixels, thermal sensitivity < 0.03 °C, a spectral range of 7.5–14 µm, and adjustable parameters for emissivity, reflected temperature, and distance correction. The emissivity value was set at 0.98, as recommended for biological tissues [31] and for cattle skin [32]. Before collecting the thermograms, environmental parameters (air temperature, relative humidity, and wind speed) were measured using an environmental datalogger (Hobo UX-100; Onset Computer Corporation, Bourne, MA, USA) and an anemometer (RS 90 PRO; RS Components Ltd., Corby, UK). Subsequently, the reflected temperature was measured to optimize the accuracy of the thermographic image. To optimize the accuracy of the thermographic images and minimize noise, a reference image of a Lambert surface was captured before each session to define radiance emission and eliminate the effect of surface reflections on the animals under study [33]. All thermographic images were collected in the home pen prior to each veterinary visit, with the calf gently restrained. For pair-housed calves, both animals remained together in the pen during measurements in order to minimize the effects of social isolation. At Farm 2, image acquisition was consistently performed in the indoor area to avoid the potential confounding effect of solar radiation. The order of image acquisition within each calf pair was randomized, and images were taken from a distance of 0.5 m at a 90° angle to each lacrimal caruncle.

### 2.5. Data Analysis

**Figure 1:**
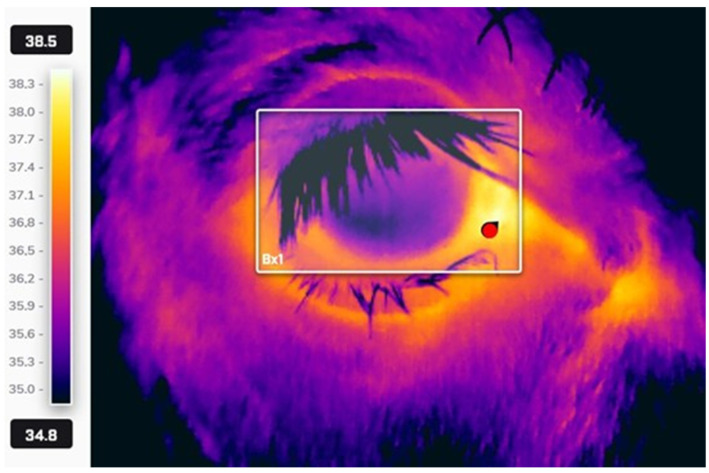
Thermogram of the right eye of one of the calves included in the study. The red mark on the lacrimal caruncle indicates the highest temperature within the rectangular area detected.

The thermograms were analyzed using the software FLIR Thermal Studio (v. 2.0.62). A specific rectangular shape subtended the lacrimal caruncle, and the maximum temperature within the selected area was calculated (Figure 1).

For each timepoint, the mean temperature of the lacrimal caruncle was calculated only when at least three adequate images per side were available. In cases where this requirement was not met, the corresponding timepoint was excluded from the analysis, although the calf remained included for subsequent or previous timepoints.

The mean left and right eye temperatures were calculated, and ocular asymmetry (ΔT °C) was calculated as the difference between the mean temperatures of the left and right eyes, following the definition previously reported by de Oca et al. [4].

Statistical analysis was performed using SPSS 29.0.1 (SPSS Inc., Chicago, IL, USA). Descriptive statistics were calculated for all variables. A linear mixed model was fitted with timepoint as a repeated effect; treatment (individual vs. pair housing), farm, and year as fixed effects; and calf ID as a random effect. At T2, Mann–Whitney tests were used to compare (i) healthy vs. sick calves and (ii) healthy and sick pair-housed calves.

## 3. Results and Discussion

**Figure 2:**
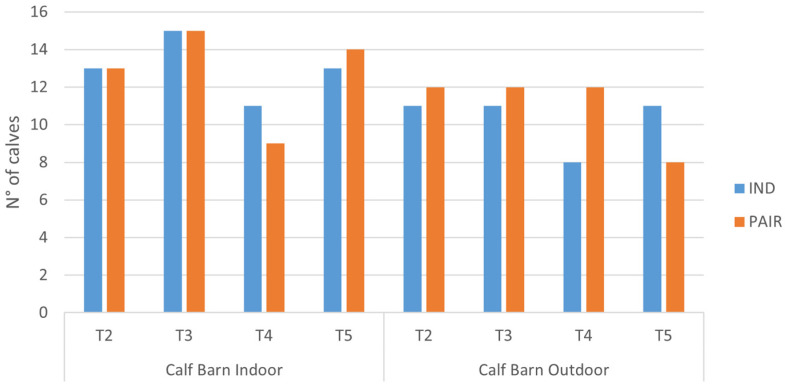
Distribution of calves with adequate thermograms in the timepoint, according to farm (Calf Barn Indoor or Calf Barn Outdoor) and individual (IND) or pair (PAIR) housing.

Following data cleaning and the exclusion of thermograms that did not meet quality criteria, a total of 188 out of 224 observations were retained, representing 83.92% of the sample. Of these, 93 corresponded to individually housed calves and 95 to calves housed in pair. Figure 2 shows the distribution of the number of animals with adequate images according to the farm and timepoint.

The main reason for the exclusion of the thermograms was the absence of the minimum number of adequate images per eye. In particular, thermograms obtained under extreme environmental conditions, such as excessive heat or during rainfall, were deemed unreliable due to the resulting alterations in image quality. Other practical limits of thermography found in the present study, such as unexpected animal movements and hair presence, were also confirmed by other studies present in the literature [14,34,35,36,37]. It is important to note that one critical aspect consider when taking thermograms for the assessment of the emotional state of animals is the need to minimize the stress induced by the practice itself. A stressful situation, in fact, such as incorrect handling or separation from the peer, can cause a rise in eye temperature caused by the activation of sympathetic nervous system [13]. For this reason, animals of the present study were restrained as gently as possible, and images were collected within the home pen of all calves. However, it cannot be fully excluded that the presence of a person within the pen might have influenced calves’ emotional state, in particular for pair-housed calves of which the thermographs were taken for second (i.e., being exposed longer to the entire procedure, from the entrance to the exit of the operator).

**Figure 3:**
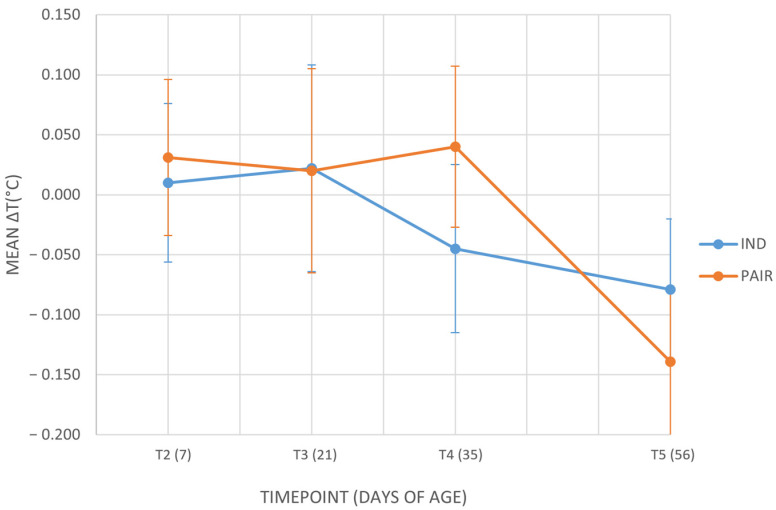
Mean ΔT values (°C) across the different timepoints in calves housed in pairs (PAIR) or individually (IND). T2 = 7 days of age; T3 = 21 days of age; T4 = 35 days of age; T5 = 56 days of age. Data are presented as mean ± standard error of the mean (SE).

Differences between left and right mean temperatures were examined: Figure 3 shows the values of ΔT across the different timepoints in calves housed in pairs or individually. Additional details are provided in the Supplementary Materials (Table S1). The mixed linear model on the outcome ΔT showed no significant effect of housing system. Estimated marginal means were nearly identical between treatments (−0.030 ± 0.035 for individually housed calves vs. −0.016 ± 0.035 for pair-housed calves; *p* = 0.773), indicating that ocular temperature asymmetry did not differ between housing conditions. Moreover, covariance parameter estimates revealed that residual variance differed significantly across timepoints (*p* < 0.001), whereas the variance attributable to calves’ identity was not significant (*p* = 0.827). This suggests that variability in ΔT was primarily associated with time rather than with individual differences.

**Figure 4:**
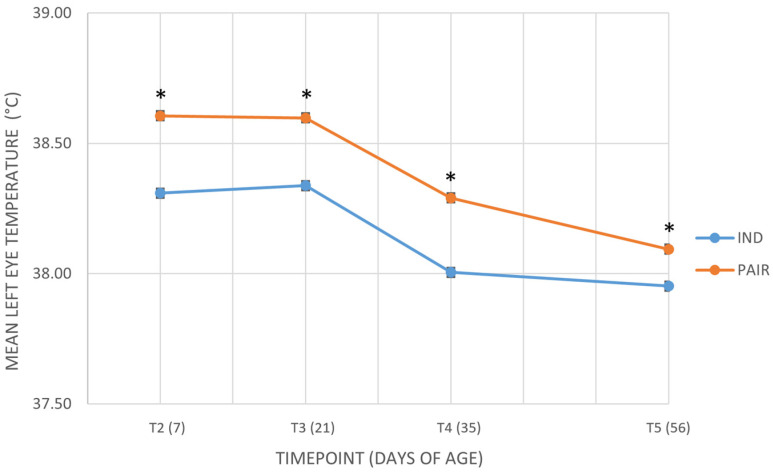
Mean left eye temperature (°C) across the different timepoints in calves housed in pairs (PAIR) or individually (IND). T2 = 7 days of age; T3 = 21 days of age; T4 = 35 days of age; T5 = 56 days of age. Data are presented as mean ± standard error of the mean (SE). An asterisk (*) indicates a statistically significant difference between individually housed (IND) and pair housed (PAIR) calves at the same time point (*p* < 0.05).

In contrast, analyses of absolute eye temperatures indicated consistent effects of housing system: Figure 4 shows the values of the mean left eye temperature (°C) across the different timepoints in calves housed in pairs or individually. Additional details are provided in the Supplementary Materials (Table S2). For the left eye, pair housed calves had slightly higher temperatures than individually housed calves (38.45 ± 0.06 °C vs. 38.19 ± 0.06 °C; *p* = 0.005). Also in this case, residual variance varied across timepoints (*p* < 0.001), while the random effect of calves’ identity was not significant (*p* = 0.104), confirming that variability was mainly explained by time.

**Figure 5:**
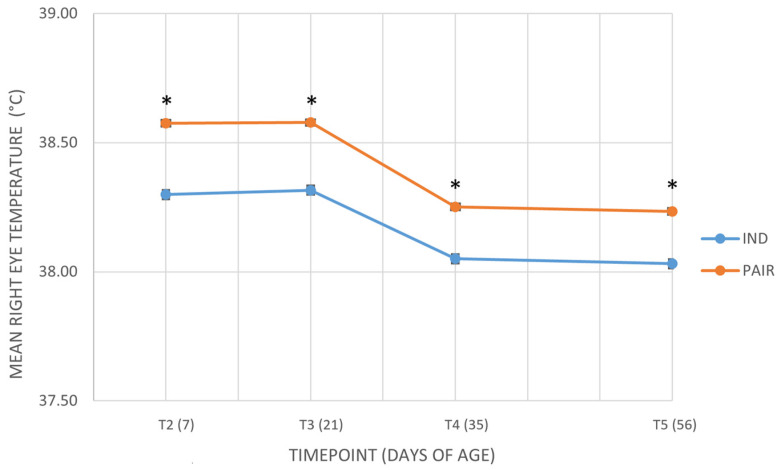
Mean right eye temperature (°C) across the different timepoints in calves housed in pairs (PAIR) or individually (IND). T2 = 7 days of age; T3 = 21 days of age; T4 = 35 days of age; T5 = 56 days of age. Data are presented as mean ± standard error of the mean (SE). An asterisk (*) indicates a statistically significant difference between individually housed (IND) and pair housed (PAIR) calves at the same time point (*p* < 0.05).

Figure 5 shows the values of the mean right eye temperature (°C) across the different timepoints in calves housed in pairs or individually. Additional details are provided in the Supplementary Materials (Table S3). Similarly, right eye temperature was significantly affected by housing, with higher values in pair-housed calves (38.41 ± 0.07 °C vs. 38.19 ± 0.07 °C; *p* = 0.026). In this case, both residual variance across timepoints (*p* < 0.001) and variance due to calves’ identity (*p* = 0.012) were significant, suggesting that variability was influenced by both time and individual differences.

Finally, farm did not significantly affect any of the three outcomes (*p* > 0.05), while the year of data collection significantly affected the mean values of the eyes (left *p* < 0.05; right *p* < 0.05) but not the delta value (*p* > 0.05).

Overall, these results do not support the lateralization hypothesis, but the decline in mean eye temperature observed across timepoints might suggest a reduced arousal of animals over time, possibly due to habituation to the procedure of image collection. As previously described, animals were restrained as gently as possible, and images were collected within the home pen of all calves the separation stress. However, we cannot fully exclude the possible influence of the presence of a person within the pen on pair-housed calves’ emotional state, in particular for the one exposed longer to the entire procedure, from the entrance to the exit of the operator. Moreover, seasonal effects may have contributed to the observed patterns. Measurements were conducted from July to November during the first sampling year, whereas data collection in the second year ended in September, thereby reducing the overlap between calf age and progressively cooler ambient conditions. Nonetheless, environmental and thermoregulatory influences on ocular temperature cannot be fully excluded, as previously reported by Reuscher et al., in particular during summertime [38,39]. Importantly, the sampling year was included in the statistical models and showed a significant effect, supporting the presence of year-specific and seasonal influences beyond calf age alone.

Pair housing may influence longer-term moods rather than momentaneous affective states. Future studies should examine positive focal events, such as calf reunion, to better explore links between housing, emotions, and brain lateralization. By contrast, research in adult cattle has largely concentrated on negative emotional contexts, particularly separation stress, consistently reporting increases ocular temperature in response to this condition [10,40]. Nevertheless, studies in ruminants (i.e., sheep) pointed out the promising use of lacrimal caruncle measurement through thermography not only for the assessment of stress and fear [41], but also to assess how caruncle’s temperature changes also in response to positive emotional states [42].

Since treatment and timepoint did not affect the lateralization indicator, a further analysis was conducted to investigate whether health status could influence this parameter. Based on the health conditions found at each veterinary visit, animals were categorized as healthy or sick. Across timepoints, the number of healthy calves consistently exceeded that of sick calves. At T2, 30 calves were classified as healthy and 19 as sick (IND: 18 vs. 6; PAIR: 12 vs. 13); at T3, 41 were healthy and 12 sick (IND: 22 vs. 4; PAIR: 19 vs. 8); at T4, 32 were healthy and 8 sick (IND: 16 vs. 3; PAIR: 16 vs. 5); and at T5, 39 were healthy and 7 sick (IND: 21 vs. 3; PAIR: 18 vs. 4). This prevented the possibility of including this factor in the statistical analysis across the four timepoints; however, a targeted analysis was performed at T2 to assess differences between healthy and sick calves, both overall and within the pair-housed group at the same timepoint. No significant differences in lacrimal caruncle temperature (i.e., ΔT, mean right and left eye temperature) were found based on health status (*p* > 0.05), neither in T2, nor in pair-housed calves at T2. Despite the limited amount of data and the preliminary nature of these findings, the lack of differences observed at T2 supports the use of lacrimal caruncle temperature as a relevant site for the assessment of emotional responses, as it appeared not to be influenced by the animals’ health status. It is possible that, under the conditions of this study, lacrimal caruncle temperature was not an indicator of either negative emotional states associated with illness or the presence of disease. Future research should explore alternative anatomical sites that might exhibit more pronounced thermal changes and assess how etiological agents and disease severity influence body temperature dynamics. Additionally, larger sample sizes and diverse environmental conditions could help refine the use of infrared thermography for assessing both emotional states and disease status in calves.

## 4. Conclusions

This study explored the potential of infrared thermography to detect positive affective states measured in pair-housed dairy calves during the pre-weaning period, compared to individual housing. Ocular temperature asymmetry (ΔT) was proposed in the literature as an indicator of brain lateralization during emotion-generating contexts. In this study pair housing was proposed as a context generating positive emotions but did not influence ocular temperature asymmetry. On the contrary, absolute eye temperatures were consistently higher in pair-housed calves compared to individually housed calves. These results suggest that pair housing might influence calves’ mood rather than punctual emotional states. Moreover, the higher eye temperatures found in pair-housed dairy calves might be associated with a higher arousal state. Importantly, in this context, changes in ocular temperature should be interpreted with caution, as variations in arousal might be influenced by procedural, environmental, and seasonal factors; moreover, in the absence of concurrent physiological or behavioral indicators, the affective valence underlying these thermal responses cannot be conclusively determined. To enable a more robust interpretation of affective states in pair-housed calves, future studies should integrate eye temperature measurements with additional physiological and behavioral welfare indicators, while also accounting for the methodological limitations highlighted in the present study. The decline in eye temperatures observed across the pre-weaning period might indicate habituation to the procedure and handling, highlighting the importance of considering time-dependent changes when interpreting IRT data. Health status at 7 days of age did not significantly affect eye temperature or asymmetry, suggesting that under the conditions of this study, the lacrimal caruncle was not a sensitive indicator of either negative affective states associated with illness or disease presence.

Overall, these findings provide novel insights into the application of IRT for calf welfare assessment and emphasize the need for further studies. Future research should focus on positive, focal events such as social reunions to better capture links between social housing, emotional valence, and brain lateralization. Moreover, exploring alternative anatomical sites, larger sample sizes, and diverse environmental contexts could further refine the use of IRT as a non-invasive tool to assess both emotional and health-related physiological responses in calves.

## Data Availability

The raw data supporting the conclusions of this article will be made available by the authors on request.

## Supplementary Materials

The following supporting information can be downloaded at https://www.mdpi.com/article/10.3390/ani16020182/s1. Table S1. Mean ΔT values (°C), standard error, and confidence interval 95% limits across the different timepoints in calves housed in pairs (PAIR) or individually (IND). Table S2. Mean left eye temperature (°C), standard error, and confidence interval 95% limits across the different timepoints in calves housed in pairs (PAIR) or individually (IND). Table S3. Mean right eye temperature (°C), standard error, and confidence interval 95% limits across the different timepoints in calves housed in pairs (PAIR) or individually (IND).
