# Supplementary material for: Through the Calf’s Eye: Exploring Infrared Thermography to Uncover Pair-Housed Calves’ Affective States
Source: Animals (Basel). 2026 Jan 8;16(2):182. doi: 10.3390/ani16020182 (PMC12837796; doi:10.3390/ani16020182)
Supplement: Supplementary file 1 [file animals-16-00182-s001.zip › animals-4034949-supplementary.pdf]

Table S1. Mean ΔT values (°C), standard error, and confidence interval 95% limits across the different timepoints in calves housed in pairs (PAIR) or individually (IND). T2 = 7 days of age; T3 = 21 days of age; T4 = 35 days of age; T5 = 56 days of age.

Housing	*Timepoint*	*Mean ΔT value (°C)*	*Standard Error (°C)*	*Confidence interval 95%, lower limit (°C)*	*Confidence interval 95%, upper limit (°C)*
IND	T2	0.010	0.066	-0.120	0.140
	T3	0.022	0.086	-0.148	0.193
	T4	-0.045	0.070	-0.183	0.093
	T5	-0.079	0.059	-0.197	0.038
PAIR	T2	0.031	0.065	-0.096	0.159
	T3	0.020	0.085	-0.147	0.187
	T4	0.040	0.067	-0.092	0.171
	T5	-0.139	0.062	-0.262	-0.017

Table S2. Mean left eye temperature (°C), standard error, and confidence interval 95% limits across the different timepoints in calves housed in pairs (PAIR) or individually (IND). T2 = 7 days of age; T3 = 21 days of age; T4 = 35 days of age; T5 = 56 days of age.

Housing	*Timepoint*	*Mean Left eye value (°C)*	*Standard Error (°C)*	*Confidence interval 95%, lower limit (°C)*	*Confidence interval 95%, upper limit (°C)*
IND	T2	38.31	0.10	38.12	38.50
	T3	38.34	0.09	38.15	38.52
	T4	38.01	0.12	37.77	38.24
	T5	37.95	0.10	37.75	38.15
PAIR	T2	38.61	0.09	38.42	38.79
	T3	38.60	0.09	38.42	38.78
	T4	38.29	0.11	38.07	38.52
	T5	38.09	0.11	37.88	38.30

Table S3. Mean right eye temperature (°C), standard error, and confidence interval 95% limits across the different timepoints in calves housed in pairs (PAIR) or individually (IND). T2 = 7 days of age; T3 = 21 days of age; T4 = 35 days of age; T5 = 56 days of age.

Housing	*Timepoint*	*Right eye Mean value (°C)*	*Standard Error (°C)*	*Confidence interval 95%, lower limit (°C)*	*Confidence interval 95%, upper limit (°C)*
IND	T2	38.30	0.10	38.10	38.50
	T3	38.32	0.11	38.10	38.53
	T4	38.05	0.11	37.84	38.26
	T5	38.03	0.11	37.82	38.24
PAIR	T2	38.57	0.10	38.38	38.77
	T3	38.58	0.11	38.37	38.79
	T4	38.25	0.10	38.05	38.45
	T5	38.23	0.11	38.02	38.45
